# Prevalence and determinants of child maltreatment among high school students in Southern China: A large scale school based survey

**DOI:** 10.1186/1753-2000-2-27

**Published:** 2008-09-29

**Authors:** Phil WS Leung, William CW Wong, WQ Chen, Catherine SK Tang

**Affiliations:** 1 Department of Community and Family Medicine, 4/F, School of Public Health, Prince of Wales Hospital, Shatin, N.T., Hong Kong, PR China; 2 Department of General Practice, Medicine, Dentistry and Health Sciences, University of Melbourne, 200 Berkeley Street, Carlton, Vic 3053, Australia; 3 Professor of Department of Biostatistics and Epidemiology, Sun Yat-sen University, 74 Zhongshan Road II, Guangzhou (510089), PR China; 4 Department of Psychology, National University of Singapore, AS4 #02-08, 9 Arts Link, Singapore 117570, Singapore

## Abstract

**Background:**

Child maltreatment can cause significant physical and psychological problems. The present study aimed to investigate the prevalence and determinants of child maltreatment in Guangzhou, China, where such issues are often considered a taboo subject.

**Methods:**

A school-based survey was conducted in southern China in 2005. 24 high schools were selected using stratified random sampling strategy based on their districts and bandings. The self-administered validated Chinese version of parent-child Conflict Tactics Scale (CTSPC) was used as the main assessment tool to measure the abusive experiences encountered by students in the previous six months.

**Results:**

The response rate of this survey was 99.7%. Among the 6592 responding students, the mean age was 14.68. Prevalence of parental psychological aggression, corporal punishment, severe and very serve physical maltreatment in the past 6 months were 78.3%, 23.2%, 15.1% and 2.8% respectively. The prevalence of sexual abuse is 0.6%. The most commonly cited reasons for maltreatment included 'disobedience to parents', 'poor academic performance', and 'quarrelling between parents'. Age, parental education, places of origins and types of housing were found to be associated with physical maltreatments whereas gender and fathers' education level were associated with sexual abuse.

**Conclusion:**

Though largely unspoken, child maltreatment is a common problem in China. Identification of significant determinants in this study can provide valuable information for teachers and health professionals so as to pay special attention to those at-risk children.

## Background

Child maltreatment results in significant medical, social, and economic costs. It has been found to be associated with a number of long-term mental health problems [1,2]. Abused children often have higher lifetime prevalence of suicide ideation and disability than the general population [3,4]. One survey conducted in Hong Kong also found that child abuse victims have a higher chance of psychiatric morbidity, more self-injurious behaviors, poorer perceived parental support and are more likely to have problems with substance abuse [5]. Despite these findings, child maltreatment has remained a taboo and hidden subject in many Asian countries like China.

Several previous studies have investigated the prevalence of child maltreatment in Chinese communities. In a recent household study conducted in Hong Kong, the prevalence of corporal punishment and physical maltreatment was 57.5% and 4.5% respectively [6]. In another study measuring the prevalence of physical abuse among Hong Kong secondary school students [7], it was reported that about 4.1% had experienced corporal punishment and 2.9% had been beaten to injury in the previous six months. A further study carried out in Beijing, China showed that 15.2% of the junior secondary school students had been beaten by parents and 56.3% of them had been scolded by parents in the previous year [8]. In terms of sexual abuse, one school-based survey among adolescents in China found that the overall prevalence of unwanted sexual experience before their age of 16 years was 13.6%, in which the prevalence was higher among girls (16.7%) than boys (10.5%) [9]. While another survey on college students in Hong Kong reported the rate of sexual abuse before the age of 17 as 6% [10].

Many studies have been aimed at identifying factors which may be associated with or are predictors of child maltreatment. For example, Berger found that violence towards children was most prevalent in single-parent families of a lower income [11]. In a longitudinal study, it was found that parents in the UK who were younger in age and with lower education level were associated with a higher likelihood of abusing their children [12]. Local evidence suggested that abusers of locally registered child abuse cases were mostly parents, aged 31–40 and housewives [13]. Consistent with western findings, they are more likely to be of education level lower than primary. Similarly, female victims and male abusers were found to be more prevalent in sexual abuse cases [10]. Such findings can play an important role in developing effective prevention strategies and programs.

Although research on child abuse is accumulating in China, large scale surveys with rigorous research design, such as stratified random sampling method used here, are not common. The present study aimed to measure the prevalence of child abuse in the region of southern China and to identify significant predictors of the abuse, which could provide important information for planning future preventive measures.

## Methods

### Procedures

In collaboration with Sun Yat-sen University in Guangzhou, this survey was conducted within high schools in Southern China between March and June 2005. In total, there were 192 high schools located in this region. All were considered eligible to join this survey and included in the sampling procedure. 24 of them were randomly selected after stratification by district (8 districts) and banding (provincial/city/district-run). Three of the invited schools refused to participate in this study for reasons unrelated to the subject under research (for example, clashes with exam timetables). As a result, another three schools were randomly selected and invited, which made up a total of 24 schools.

For each participating school, two classes from Year 7 to 9 (age 13–15 years) were randomly chosen, resulting in a total of 144 classes, with an average class size of about 46 students). The questionnaire was self administered by the students inside the classroom, but outside class hours. School principals were asked for permission to recruit students. During data collection, consent was obtained from individual students after they had been told that their participation was completely voluntary in nature, and that they could discontinue their involvement at any time. Anonymity and confidentiality of responses was also stressed, with assurances that even teachers would not be given access to the data. Our study was approved by the Survey and Behavioral Ethics Committee, the Chinese University of Hong Kong.

### Measures

A validated Chinese version of parent-child Conflict Tactics Scale (CTSPC) [14,15] was used as the main assessment tool in the present study (Appendix 1). CTSPC is suitable for use in a self-administered format among child respondents [14], which have been used to measure not only psychological and physical maltreatment of children, but also non-violent modes of discipline administered by parents. Examples of non violent behaviors included 'parents put you in time-out' and 'take away privilege'. The items for *psychological aggression *included 'threatened to spank or hit you but did not actually do it' and 'shouted, yelled, or screamed at you'.*Corporal punishment *included 'slap you on the hand, arm or leg' and 'hit you on the bottom with something like a belt, hairbrush, a stick or some other hard object'. *Severe physical maltreatment *included 'slap you on the face or head or ears' and 'threw or knocked you down', while *very severe physical maltreatment *included 'grab you round the neck and choke you' and 'burn or scald you on purpose'. Students were asked the frequency of encountering the listed behaviors in the previous six months using a 3-point Likert scale (never vs sometimes vs often). Their responses would be classified as indicating "maltreatment of a particular type" if they had experienced any one or more of the depicted behaviors within the corresponding subscale.

Whenever the students had experienced physical maltreatment, they were further asked to indicate the reasons for the maltreatment. Numerous possible reasons were listed, which could be classified into two main types. The first type was concerning their own misbehaviors, such as 'did poorly academically', 'break school rules', 'disobey to parent', 'stealing', 'misbehave in public' and 'hit or fight with siblings or playmates'. The second types related to the problems of the parents, including 'parents disputed', 'parent drunk/drugged' and 'parents lost money due to gambling'. Students were asked to write down any reasons if not listed above.

Another two items were used to measure the previous parental sexual abuse experience, namely 'parents fondle your breasts or genital or sex organs' and 'parents ask you to fondle their genital or sex organs' [10]. Students were asked to indicate whether they had experienced these situations in the previous six month using a three-point response scale (never vs. sometimes vs. always). In addition, other variables measured included age, gender, place of origin (Guangdong vs. non-Guangdong), housing type (lived in house owned by family vs. rented house vs. institution hostel) and level of parental education.

### Data analysis

Data was entered with the statistical program Epidata, which was then cleaned and analyzed using SPSS (version 13). Frequency of various maltreatments was reported, while descriptive details like mean and standard deviations (SD) were also obtained. In order to calculate the crude odds ratios (OR) and 95% confidence intervals (95%CI) in bivariate association, demographics of respondents and the occurrence of maltreatment were dichotomized and their relationships examined by chi-square tests. Since corporal punishment was found to be a risk factor for maltreatment [16] and identifying its determinants could also be useful for the prevention of child maltreatment, it was also classified as a physical maltreatment group together with the severe and very severe maltreatment subgroups.

Logistic regression models were also conducted to measure the independent effect of different variables on the occurrence of maltreatment. All the possible determinants including age, gender, place of origin, housing type and parental education level were controlled for in the model before the odds ratios of each variable obtained.

## Results

Most students agreed to participate in the survey. Of the 6649 questionnaires administered, 6628 questionnaires were completed yielding a response rate of 99.7%. 36 of them were excluded for further analysis due to excessive missing or invalid data. The demographic details of the remaining 6592 students are shown in Table 1. Their mean age was 14.68 (SD = 1.00). There were approximately equal numbers of males and females (50.1% vs. 49.9%). More than half of them (59.0%) aged 14 or below. The majority had their place of origin in Guangdong province (85.5%), and with parents' education levels higher than primary school level (fathers: 94.5%; mothers: 89.2%). The majority of them (78.6%) were living in a property owned by the family, with the proportion of those living in rented accommodation representing the next largest group (14.3%).

**Table 1:** Demographics of responding students

		n	%
Gender	Male	3277	49.9
	Female	3285	50.1

Age	13 or below	804	12.4
	14	1941	30.0
	15	2082	32.2
	16 or above	1637	25.3

Father's education level	Primary or below	357	5.5
	Lower secondary	1953	30.2
	senior secondary	2807	43.5
	Tertiary or above	1340	20.7

Mother's education level	Primary or below	697	10.8
	Lower secondary	2189	33.8
	senior secondary	2461	38.0
	Tertiary or above	1132	17.4

Place of origin	Guangdong	5193	85.6
	Non-Guangdong	877	14.4

Housing type	Own house	5161	78.6
	Rented house	936	14.3
	Institution hostel	376	5.7
	Others	91	1.4

Prevalence of abuse	Psychological aggression	5192	78.3
	Corporal punishment	1538	23.2
	Severe physical maltreatment	1001	15.1
	Very severe physical maltreatment	186	2.8
	Sexual abuse	41	0.6

In the previous six months, 78.3% (5192) of the students had experienced psychological aggression (verbal abuse or threats) from their parents; 23.2% (1538) of them reported corporal punishment; 15.1% (1001) reported severe physical maltreatment and 2.8% (186) reported very severe physical maltreatment. Among the students who have suffered corporal punishment or physical maltreatment, 66 (3.8%) and 23 (1.3%) of the 1767 victims were so severe that they had required medical attention and hospital admission respectively. For all types of maltreatment, the most commonly cited reasons for psychological aggression and physical abuse were 'disobedience to parents' (ranging from 34.4% to 40.2% across different forms of abuse) and 'poor academic performance' (29.6%–43.0%), followed by 'quarreling between parents' (5.3%–8.1%). For sexual abuse, the overall prevalence was found to be 0.6% (41 students).

Table 2 and 3 showed the factors associated with the occurrence of physical maltreatment and sexual abuse respectively. Age was dichotomized at the cut-off point at 14 years old using the mean age. Chi square tests showed that those who aged 14 years or below were about 1.52 times (95%CI 1.35–1.69) more likely to have experienced physical maltreatment (including corporal punishment, severe or very severe assault); while those from Guangdong province (OR 0.68; 95%CI 0.58–0.79) and living in their own house (OR 0.75; 95%CI 0.66–0.85) were less likely to have such experience. Similarly, parental education level was also dichotomized at 'primary school or below', since a previous study had shown that a significant proportion of abusers were below this level of education [13]. Our results showed that having parents of education level lower than secondary was significantly associated with higher chance of maltreatment among the students (father: OR 1.30; 95%CI 1.03–1.64; mother: OR 1.19; 95%CI 1.00–1.41). Logistic regression models demonstrated that age (OR 1.49; 95%CI 1.32–1.68), place of origin (OR 0.71; 95%CI 0.61–0.84) and housing type (OR 0.79; 95%CI 0.68–0.91) remained significantly associated with the occurrence of maltreatment after adjusting for other variables.

**Table 2:** Determinants of physical maltreatment among the responding high school students

		Physical maltreatment		Crude OR	Adjusted OR^#^
		No	Yes	(95%CI)	(95%CI)
Gender	Male	2376 (72.5%)	901 (27.5%)	1.08	1.07
	Female (refcat)	2428 (73.9%)	857 (26.1%)	(0.96–1.20)	(0.95–1.68)

Age	14 or below	2668 (69.8%)	1152 (30.2%)	1.52	1.49
	15 or above (refcat)	2057 (77.8%)	587 (22.2%)	(1.35–1.69)*	(1.32–1.68)*

Father's education level	Primary or below	243 (68.1%)	114 (31.9%)	1.30	1.15
	Secondary or above (refcat)	4484 (73.5%)	1616 (26.5%)	(1.03–1.64)*	(0.89–1.50)

Mother's education level	Primary or below	488 (70.0%)	209 (30.0%)	1.19	1.10
	Secondary or above (refcat)	4253 (73.6%)	1529 (26.4%)	(1.00–1.41)*	(0.90–1.33)

Place of origin	Guangdong	3869 (74.5%)	1324 (25.5%)	0.68	0.71
	Non-Guangdong (refcat)	583 (66.5%)	294 (33.5%)	(0.58–0.79)*	(0.61–0.84)*

Housing type	Own house	3844 (74.5%)	1317 (25.5%)	0.75	0.79
	Rented house/institution hostel/others (refcat)	962 (68.6%)	441 (31.4%)	(0.66–0.85)*	(0.68–0.91)*

* significant at 0.05 level

^# ^adjusted odd ratios was calculated in logistic regression model with the control for other risk factors listed in the table

**Table 3:** Determinants of sexual abuse among the responding high school students

		Sexual abuse		Crude OR	Adjusted OR^#^
		No	Yes	(95%CI)	(95%CI)
Gender	Male	3249 (99.1%)	28 (0.9%)	2.86	1.97
	Female (refcat)	3275 (99.7%)	10 (0.3%)	(1.37–5.88)*	(0.91–4.24)

Age	14 or below	3797 (99.4%)	23 (0.6%)	1.22	1.62
	15 or above (refcat)	2631 (99.5%)	13 (0.5%)	(0.62–2.44)	(0.74–3.57)

Father's education level	Primary or below	350 (98.0%)	7 (2.0%)	3.85	3.24
	Secondary or above (refcat)	6069 (99.5%)	31 (0.5%)	(1.72–9.09)*	(1.03–10.21)*

Mother's education level	Primary or below	692 (99.3%)	5 (0.7%)	1.27	1.69
	Secondary or above (refcat)	5749 (99.4%)	33 (0.6%)	(0.49–3.22)	(0.46–6.25)

Place of origin	Guangdong	5169 (99.5%)	24 (0.5%)	0.67	0.89
	Non-Guangdong (refcat)	871 (99.3%)	6 (0.7%)	(0.28–1.65)	(0.34–2.38)

Housing type	Own house	5137 (99.5%)	24 (0.5%)	0.50	0.61
	Rented house/institution hostel/others (refcat)	1390 (99.1%)	13 (0.9%)	(0.25–0.98)*	(0.27–1.37)

* significant at 0.05 level

^# ^adjusted odd ratios was calculated in logistic regression model with the control for other risk factors listed in the table

Unlike physical maltreatment, sexual abuse was significantly related to the gender of students. However, the relationship was unexpected, in that the male students were three times more likely to be abused than the female students (OR 2.86; 95%CI 1.37–5.88). We found that those who were living in family owned premises were less likely to have experienced sexual abuse (OR 0.50; 95%CI 0.25–0.98). Moreover, students whose fathers had educational level of primary or below were 3.85 times (95%CI 1.72–9.09) more likely to have experienced sexual abuse, with this association remaining significant in logistic regression models (OR 3.24; 95%CI 1.03–10.21).

## Discussion

The results of our study suggest that child maltreatment in China is, despite the lack of cultural recognition of such problems, a relatively widespread phenomenon. Nearly four-fifths of the responding students indicated that they had experienced psychological aggression. Corporal punishment was reported by a quarter of the students, more than 15% of whom had experienced severe physical maltreatment. Factors such as age, gender, low parental education level and having been born outside Guangdong province were found to be associated with the occurrence of maltreatment.

This study represents one of only a few large scale, school based studies that have investigated the issue of child maltreatment in southern China, and a particular strength of this study is the high response rate that was successfully achieved in this setting. Moreover, the use of stratified random sampling strategy suggests that the results are likely to be representative of the population at large, which allows a reasonably accurate estimation of prevalence and risk factors associated with child maltreatment among the intended population in this region. This study has an added advantage in that a validated tool was adopted. For example, corporal punishment was simply measured by one question of whether the respondents were ever beaten in one Beijing study [8]. However, there are a few limitations: this study was based on high school students only and thus, there may be a limit to the generalizations that can be drawn to those outside the mainstream educational system: however, previous research suggests that the prevalence of child maltreatment in such communities is likely to be greater than that reported here. In addition, as this study was a cross sectional study, no causal relationship between variables can be drawn.

It has been suggested that the definition and perception of child maltreatment is typically shaped by culture norms [17]. Tang has suggested that under the Chinese culture, children are expected to obey to their parents, while parents will apply strict disciplines such as corporal punishment to ensure their children's loyalty and respect [13]. The findings of the present study were comparable with other studies conducted within the Chinese community such as China and Hong Kong, which is only 130 km (80.8 miles) away from Guangzhou and exhibits many similarities in cultural and social norms.

In a recent Hong Kong study, the prevalence of corporal punishment and physical maltreatment were measured by the self-reported response of parents [6]. It was found that among those parents whose children aged between 13- and 17-years-old, the rate of corporal punishment by mother and father were 28.3% and 13.6% for male children, and 31.0% and 22.7% for female children respectively. These figures were comparable with the present study (23.2%). In terms of maternal and paternal physical maltreatment, the prevalence was 2.7% and 1.1% for male children, and 4.0% and 2.3% for female children. These lower rates of physical maltreatment (compared to 15.1% in the present study) could be attributed to different study designs in which parents instead of the children were chosen as the respondents, and possible differences in how they perceived such behaviors. It is possible that although parents were willing to disclose their use of corporal punishment, they were reluctant to admit the true frequency of more violent behaviors that they may exhibit. On the other hand, the prevalence of sexual abuse was also lower than that found in a previous study (6%) [10], possibly because this study looked at sexual abuse by parents only.

There has been contradictory evidence regarding the relationship between the age of the children and maltreatment. The results of the present study supported that physical maltreatment was more prevalent among children in younger age, giving support to a previous findings by Lau [18]. However, it was surprising to find that male students were about 3 times more likely to have experienced sexual abuse than girls. This result is difficult to explain. It is widely believed that boys are much more reluctant than girls to disclose their experience of sexual abuse because of the gender stereotype that males are of stronger character. It is possible that in the present study, the school setting provides a safe environment which allows these youths to be more willing to disclose their true experiences.

Many previous studies conducted in foreign countries have shown that lower parental educational level was associated with a higher likelihood of child physical maltreatment [12,18]. Reviewing literature from the Chinese community fails to confirm such a relationship with both physical maltreatment [5,16] and sexual abuse [8,19], with an exception that one study of all registered child abuse cases in Hong Kong reported 60% of the perpetrators were educated to primary level or below. This study adds support to the international findings and implies that focusing on education and general quality of life may be an effective means in reducing child maltreatment in the future.

Students who were not born in Guangdong province were found to be more likely to have experienced physical maltreatment. This result provided additional supporting evidence to previous findings that lower income and parents' poor social network were risk factors for child maltreatment [12]. It is believed that most of these families came from their home town in order to find a better paid job in the more prosperous Guangdong province. On the one hand, they are more likely to be hired in a low-paid job and in unsatisfactory financial condition. On the other hand, since most of their friends and relatives remain in their hometown, these 'immigrant' families may have a poorer social network. Our results therefore suggest that these poorer migrant families may be an appropriate target for prevention strategies.

Child maltreatment, especially for physical abuse, was shown to be more common among students whose fathers were of lower education levels (compared to marginal association with mother's education) and who were not living in premises that they owned, while the two most common reasons for maltreatment were 'disobedience to parents' and 'poor academic performance'. One possible interpretation was that among the families of lower socio-economic status, the expectation and frustration related to improving the future living conditions of the parents and the families will have fallen on the next generation, and generates strict disciplinary methods. It is common in the Chinese community for strict discipline to be adopted to correct any 'inappropriate behaviors', for example, when not performing well at school, which can eventually escalate to severe physical maltreatment. One previous study in Hong Kong has provided partial support for this notion, finding that physical maltreatment was more likely to occur among children who were blamed for their poor academic performance by parents [7]. However, the interactive effects of low socio-economic status and children's poor educational accomplishment on child maltreatment within the Chinese culture would require further research to validate.

## Conclusion

Child maltreatment is a serious and common problem in China. This study found that among the responding students, the prevalence of psychological aggression, corporal punishment, severe physical maltreatment and very severe physical punishment were 78.3%, 23.2%, 15.1% and 2.8% respectively. Furthermore, students who were male, of younger age, moved from other places and of lower parental education level were identified as being at significant risk of maltreatment. More attention should be paid by health professionals and teachers to these children.

## Abbreviations

SPSS: Statistical package for the social sciences software.

## Competing interests

The authors declare that they have no competing interests.

## Authors' contributions

LWS responsible for the coordination of the project, data analysis, interpretation of the data and the drafting of manuscript. WCW has involved in the conception and design of the study, interpretation of the data and revising the manuscript critically for important intellectual content. CWQ conducted the data collection in Guangzhou, China. TSK has involved in the conception and design of the study and revising the manuscript. All authors have read and approved the final manuscript.

## Appendix

Appendix 1 Items measured the physical maltreatment among responding students

**Table 4:** Items measured the physical maltreatment among responding students.

**Non-violent behaviors**
Your parents put you in "time out" (or send you to your room).
Your parents take away privileges or ground you?
Your parents threaten to spank or hit you but did not actually do it.
Your parents shout, yell or scream at you.
Your parents swore or curse at you.
Your parents call you dumb or lazy or some other names like that.
Your parents say that they would send you away or kick you out of the house.
**Corporal punishment**
Your parents spank you on the bottom with their bare hands.
Your parents hit you on the bottom with something like a belt, ruler, a stick, sweeper or some other hard object.
Your parents slap you on the hand, arm, or leg.
Your parents pinch you.
Your parents shake/push you.
**Severe assaults**
Your parents slap you on the face or head or ears
Your parents hit you on some other part of the body besides the bottom with something like a belt, ruler, a stick, sweeper or some other hard object
Your parents throw or knock you down
Your parents hit you with a fist or kick you hard
**Very severe assaults**
Your parents beat you up, that is they hit you over and over as hard as they could.
Your parents grab you around the neck and choke you.
Your parents burn or scald you on purpose.
Your parents threaten you with a knife or other weapons.
Your parents immerse your head in water or other inflicted injury with sharp objects, e.g. knife or broken glasses.
